# Plasticity Regulators Modulate Specific Root Traits in Discrete Nitrogen Environments

**DOI:** 10.1371/journal.pgen.1003760

**Published:** 2013-09-05

**Authors:** Miriam L. Gifford, Joshua A. Banta, Manpreet S. Katari, Jo Hulsmans, Lisa Chen, Daniela Ristova, Daniel Tranchina, Michael D. Purugganan, Gloria M. Coruzzi, Kenneth D. Birnbaum

**Affiliations:** 1 Center for Genomics and Systems Biology, Department of Biology, New York University, New York, New York, United States of America; 2 School of Life Sciences, University of Warwick, Coventry, United Kingdom; 3 Warwick Systems Biology Centre, University of Warwick, Coventry, United Kingdom; 4 Department of Biology, The University of Texas at Tyler, Tyler, Texas, United States of America; 5 Greater Baltimore Medical Center, Baltimore, Maryland, United States of America; 6 Courant Institute of Mathematical Sciences, New York University, New York, New York, United States of America; Harvard University, United States of America

## Abstract

Plant development is remarkably plastic but how precisely can the plant customize its form to specific environments? When the plant adjusts its development to different environments, related traits can change in a coordinated fashion, such that two traits co-vary across many genotypes. Alternatively, traits can vary independently, such that a change in one trait has little predictive value for the change in a second trait. To characterize such “tunability” in developmental plasticity, we carried out a detailed phenotypic characterization of complex root traits among 96 accessions of the model *Arabidopsis thaliana* in two nitrogen environments. The results revealed a surprising level of independence in the control of traits to environment – a highly tunable form of plasticity. We mapped genetic architecture of plasticity using genome-wide association studies and further used gene expression analysis to narrow down gene candidates in mapped regions. Mutants in genes implicated by association and expression analysis showed precise defects in the predicted traits in the predicted environment, corroborating the independent control of plasticity traits. The overall results suggest that there is a pool of genetic variability in plants that controls traits in specific environments, with opportunity to tune crop plants to a given environment.

## Author Summary

Plants can dramatically alter their development in order to cope with new environmental conditions. Such plasticity is especially evident in the root system since it adopts a particular architecture under one condition, but can change architecture by altering the extent of lateral root branching in a different condition. To explore the extent of root plasticity to the critical nutrient nitrogen we analyzed a natural population of the model plant *Arabidopsis* in both nitrogen-limiting and nitrogen-rich environments. This revealed that root architecture plasticity appears to be the combined effect of many individual root responses to the environment that are independently modulated. Each aspect, such as lateral root length, number, or density seems to be turned on or off separately, giving the whole system flexibility. We then identified specific genes that control these individual component responses by exploring the genetic variation across the natural population in combination with analyzing which genes respond to nitrogen. Together the results help us gain insights into how the environment shapes plant development. This knowledge can be used to better understand how the growth of our existing crop species might change as the climate varies, and identify new crop varieties that will be robust to such variation.

## Introduction

Nitrogen is a limiting nutrient in plant growth that is typically taken up from the soil by the root system [1]. However, because the soil environment often varies over space and time, a single genotype needs to adjust its root architecture in response to different soil conditions, an example of developmental plasticity. One imperative in agriculture is to develop crops that can grow efficiently while reducing expensive and environmentally detrimental nitrogen supplements; current high yield crops are typically optimized for a single environment of high nitrogen. To breed crops for different or naturally fluctuating nitrogen environments, mechanisms that mediate traits conditioned on the environment may be important targets of crop improvement.

In plants, root architecture is a complex phenotype that arises from adult meristematic activity in primary and lateral roots and lateral root initiation [2], [3]. These traits collectively determine the root's three-dimensional body plan, where specific shapes can provide advantages in certain environments [4]. For example, deeper primary roots are often associated with plants with a greater tolerance to drought [5], [6]. The dynamic and patchy nature of the soil environment also appears to make the post-embryonic adjustment of the body plan a valuable attribute. For example, a strong association was found between local proliferation of lateral roots and nitrogen uptake in competition assays in grasses [7], [8]. Collectively, these studies show that different attributes of root architecture and the ability of individuals to adjust that architecture can confer advantages in the heterogeneous soil environment.

Here, we systematically characterize the way in which root traits can vary in different environments across accessions in one species, *Arabidopsis thaliana*. At one extreme, a set of traits may be correlated (or anti-correlated) such that trait 1 and 2 may both consistently increase, decrease or show opposite trends in a new environment when examined in many different accessions [7]. At the other extreme, traits may be independent with respect to each other, such that a change in trait 1 has no predictive value in a change in trait 2 when examining many genotypes [4].

We expect that specific genes mediate the response to extrinsic signals to affect intrinsic development programs [2]. For example, genes that mediate the activation of transient stem cell niches in the pericycle will influence lateral root density [4]. In previous work, plasticity mechanisms active in the pericycle were found to coordinate the control of root initiation and outgrowth by nitrogen. It was found that an increase in expression of the transcription factor *Auxin Response Factor 8* in response to high nitrogen treatment decreased lateral root growth and increased lateral root initiation [9]. This was an example of trait coupling that could result in an anti-correlation between traits across many genotypes. Another study showed that C∶N ratio appeared to specifically control lateral root initiation without strongly influencing other root traits – a potential example of trait independence [10]. A few other cases of regulatory genes that control root architecture in response to nitrogen have been identified [11], [12]. However, the overall level of customization of phenotype to environmental variation and the genetic architecture underlying plasticity are not well understood.

To characterize the tunability of root traits in response to different environments, we merge concepts from two different fields. The field of phenotypic integration has documented the level of correlation vs. independence in traits, typically across different genetic variants within a species or a set of closely related species [13]. The field of phenotypic plasticity has documented the ability of single genotypes to show variable phenotypes in different environments [13]–[15]. Here, we examine the correlation vs. independence of the difference in root traits in two environments to ask how finely the plant can manipulate its developmental plasticity. In addition, we also seek to determine the genetic mechanisms that mediate plasticity, as there is growing interest in the genes underlying phenotypic plasticity [13], [16], [17].

By carrying out comparative phenotypic analysis of key features of root architecture, we have been able to both analyze the correlation of individual root traits as well as assess the extent of plasticity within and between root traits that form root architecture. The use of genomic expression profiling in combination with high-density genetic marker association analysis enabled identification of genes implicated in controlling independent root parameters. By combining the phenotypic and genomic analyses we were able to functionally validate a number of new root regulators that mediate the response to nitrogen levels in discrete environments.

## Results and Discussion

### Root traits display a high degree of independence across nitrogen environments

We characterized the level of correlation vs. independence in root plasticity by asking how traits vary in two distinct nitrogen environments among 96 well-characterized natural accessions or ecotypes of the model species *Arabidopsis thaliana* [18] (see Materials and Methods). We define developmental plasticity as the ability of a single genotype to exhibit different phenotypes in different environments. If root trait differences in the two environments are correlated, accessions should exhibit similar suites of changes in root architecture in distinct nitrogen environments. Alternatively, if a high level of trait independence exists, genotypes that are similar in one nitrogen environment could alter a subset of root traits in a new nitrogen environment.

**Figure 1:**
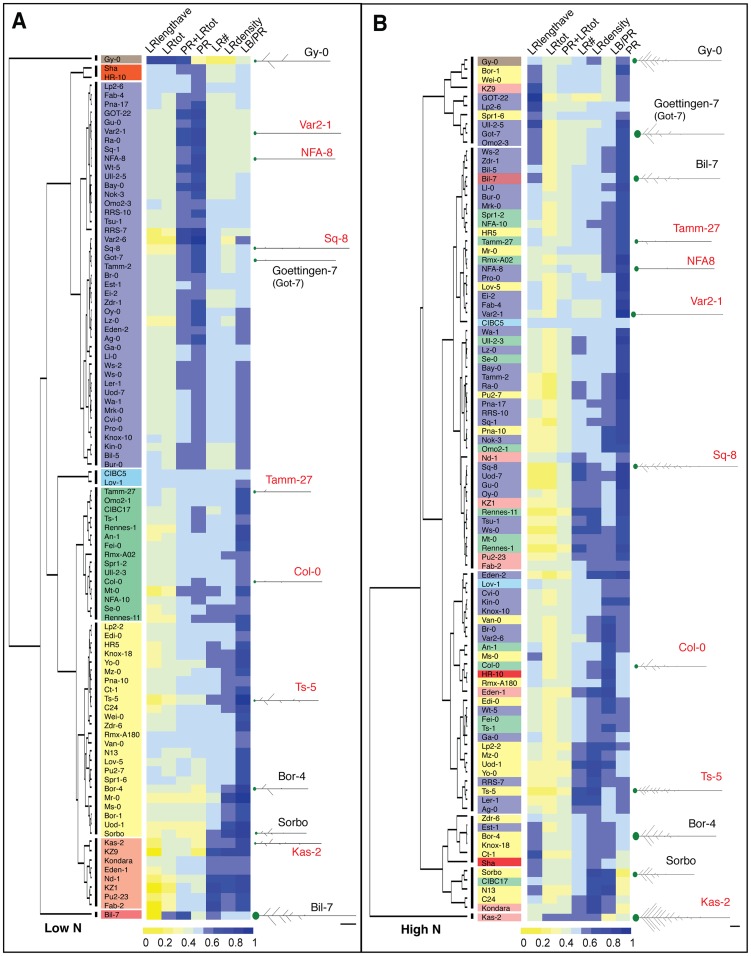
Clustering of 96 accessions grown on high nitrogen and low nitrogen based on root traits. (A) Clustering based on low N traits (scaled) forms eight clusters, which are indicated by vertical lines next to the dendrogram. Accessions within correlated groups (clusters) are highlighted in different colors. (B) Clustering based on high N traits (scaled) forms six clusters. The cluster designations in low N are highlighted in the same color as used in (A), illustrating the alternate topologies in different environments. (A–B) Adjacent to each dendrogram is a heatmap visualizing the scaled trait values for each ecotype; see color scale bar for values. Several examples of average seedling root architecture are shown for the seven accessions chosen for expression analysis (red text) and five additional accessions to illustrate root architecture in each cluster. Scale bars = 1 cm.

**Figure 2:**
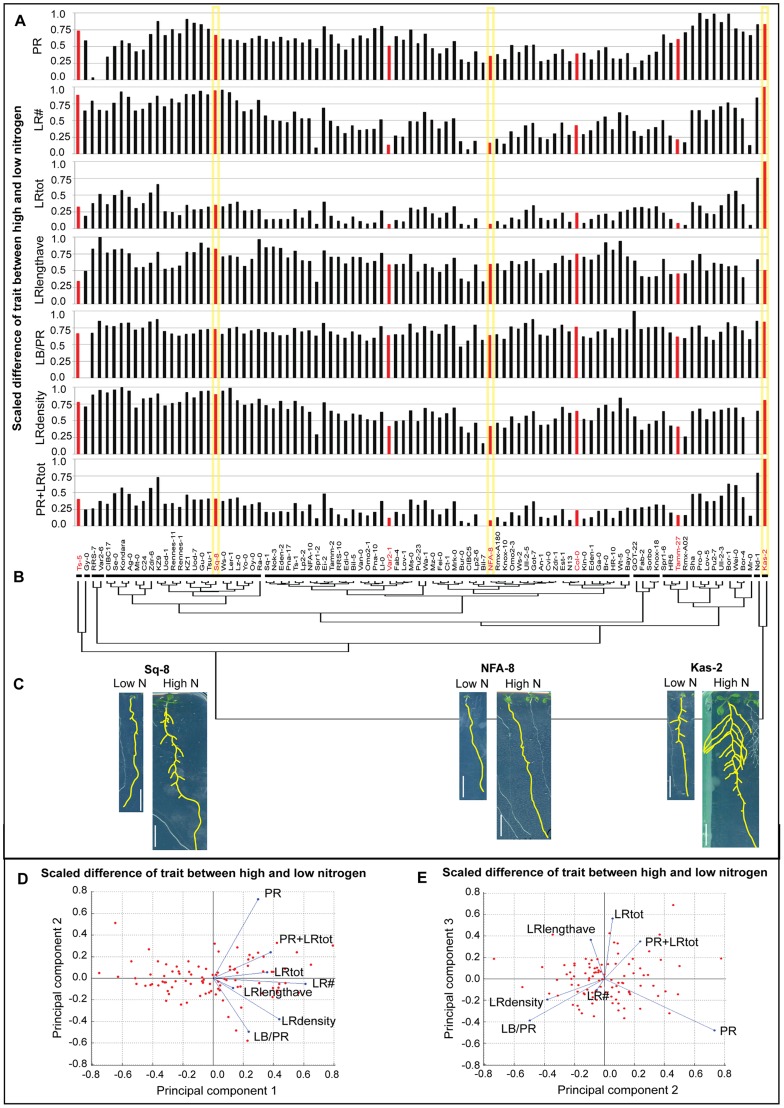
Changes in root traits among natural variants in two different nitrogen environments. The difference between root trait values on low and high nitrogen (δ highN-lowN) is represented in the form of individual bar charts (A) and trait differences were used to form a dendrogram of accessions resulting in nine clusters as indicated by horizontal lines (B); the seven accessions chosen for expression analysis are highlighted in red. (C) Images of low N and high N grown seedlings from representative accessions of a ‘strong N-responder’ Sq-8, a ‘weak N-responder’ NFA-8, and the ‘super N-responder’ Kas-2 with yellow highlighting on their trait difference values on (A); scale bars = 1 cm. (D–E) PCA analysis of the δ highN-lowN for the seven accessions. Red markers indicate position of 96 accessions as determined by their trait values in the given principal components. Blue lines represent vectors that quantify the magnitude and direction of a trait's contribution to that axis. For example, in (E), an accession with a high score in PC3 will have a high LRtot value. PC2 is not informative for LRtot but accessions with a long primary root (PR) will score highly on this axis. The percent variability explained by PCs 1, 2 and 3 are 64%, 17%, and 10%, respectively.

To address this hypothesis, we first clustered accessions based on seven root traits capturing root size and architecture (see Methods) in each of the two environments: high and low nitrogen (Figure 1). There was a dramatic rearrangement of the tree topology in the two environments, as shown by the dispersal of clusters formed in low nitrogen mapped onto the high nitrogen phenotype tree (Figure 1). To observe trait behaviors, we also clustered accessions based on their trait differences in the two nitrogen growth conditions, and mapped average trait differences in each accession onto the tree as bar graphs (Figure 2A,B) or a heatmap (Figure S4). In one example, NFA-8 and Sq-8 have similar architectures on low nitrogen, but exhibit much different phenotypes in the high nitrogen environment, where Sq-8 outgrows lateral roots much more dramatically (Figure 2C). In another clade, Kas-2 is a super-responder, dramatically increasing almost all root traits measured in high nitrogen to the extreme levels observed (Figure 2C). On the other hand, roots of Bil-7 are almost completely unresponsive to nitrogen (Figures 1,2A). Overall, the cluster analysis indicates that sharing a phenotype in one nitrogen environment does not predict similarity in root architecture in a second nitrogen environment, arguing that different trait responses are independent of one another.

### Independence of root traits underlies plasticity responses to nitrogen

We used a Principal Components Analysis (PCA) to investigate the degree of correlation vs. independence in the root traits. In the first PC, which accounted for 64% of variation, almost all traits showed the same magnitude and direction in their contribution (Figure 2D, blue lines). This trend suggested that the greatest variation among the difference of traits on high compared to low nitrogen were correlated changes in traits, meaning overall size differences (Figure 2D). However, the different traits made highly varied contributions to the second and third PCs, as shown by the vectors (blue lines) representing the magnitudes and sign of trait coefficients in each component (Figure 2E). The second and third components represented about 17% and 10% of the variance, respectively, indicating a substantial amount of variation in these two components. Interestingly, the star-shaped configuration of the coefficient vectors indicates that traits are highly orthogonal in the space of the second and third principal components. In other words, traits show a high degree of independence and lack of correlation. The same trends were found in a PCA analysis of trait data from high or low nitrogen conditions or the combined high plus low nitrogen dataset (Figure S5). The substantial variation in PCs 2 and 3 shows that there is a significant component of variation in which traits vary freely among accessions in the transition from one environment to another.

Similarly, a mixed model ANOVA of the trait data showed that almost all traits have accession-by-treatment interactions (see Methods). For example, in the ANOVA model, Kas-2 has a high interaction coefficient in LRtot, in which it changes phenotype dramatically in the two nitrogen conditions (Figure 2C). In a different type of trait interaction, Kas-2 also has a high interaction coefficient in L_B_/PR (root length between hypocotyl and most distal lateral root), but it is one of the few accessions to show almost no phenotypic difference between nitrogen environments (Table S5). Overall, the analysis shows that individual accessions adjust to differing nitrogen environments with variable increases in overall size, which demonstrates trait correlation as in PC1. However, there is a prominent secondary source of variability in which traits vary independently among the accessions and between environments, as demonstrated in PCs 2 and 3. The result shows that much of the variation observed when growing the 96 accessions in two environments is comprised of overall size effects, but, importantly, another large component of variation reflects a high level of fine tuning of each accession to a particular nitrogen environment.

### Distinct sets of genes associate with specific root traits in the two environments

To identify mechanisms involved in plasticity, we employed a genome-wide association study (GWAS) [19]. We associated known SNPs with root traits from plants grown on low or high nitrogen environments, or the difference in a trait value between the two environments (Table S6). In addition, we calculated the total proportion of trait heritability that the SNPs explain (Tables S6,S7). We used 96 accessions, as previous work suggested this number is sufficient to identify associations with relatively strong effect [19]. In total, we found 53 highly significant SNP hits that could be grouped, based on proximity, into 17 SNP groups (a SNP window included all genes within 10 kb on either side of the SNP and such intervals were joined into “groups” if their windows overlapped). In total, the 17 SNP groups encompassed 106 genes (Table S8). Surprisingly, out of 17 SNP groups, only a third of the groups associated with the same trait in the two nitrogen environments. This could mean that we either lacked power to detect SNPs in one environment, or, that there is genetic variation that specifically influences phenotype in one environment. We sought to test the hypothesis that specific genes mediate distinct traits in one nitrogen environment by testing whether mutants in any of the genes found within intervals showed a phenotype in the associated trait in the predicted environment. We focused on lateral root average length because 7 SNP groups encompassing 53 genes showed high significance and because a number of insertional mutants were available for genes in these windows (Table S8).

### Transcriptional changes to nitrogen influx corroborate genetic associations

**Figure 3:**
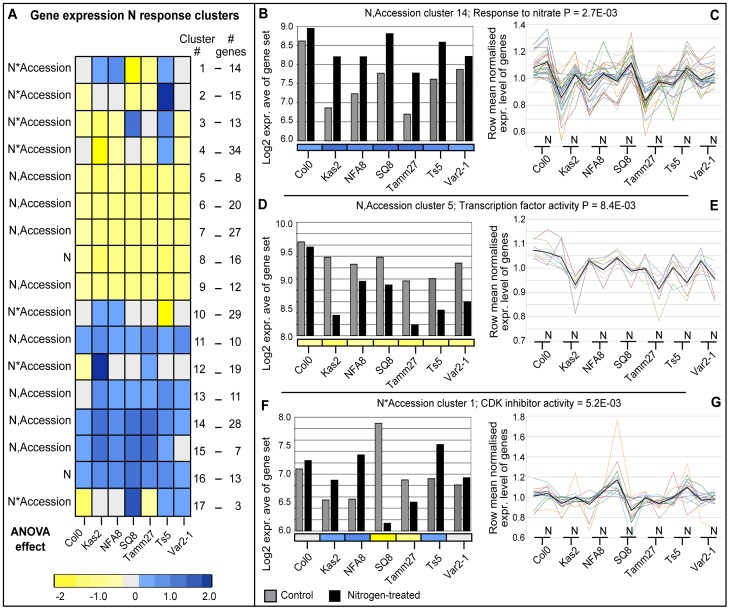
Global expression analysis of genes differentially regulated by nitrogen across representative accessions. (A) A heatmap showing cluster patterns of N responses across accessions ordered by clustering on Euclidean distance; see color scale bar for log2 fold change magnitude and direction. Among N*Accession genes, the GO terms ‘response to stimulus’ (30 genes, *P* = 8.5E-03), and ‘RNA binding’ (9 genes, *P* = 8.1E-03) were both overrepresentated; BioMaps GO term overrepresentation analysis. (B,D,F) Average log2 microarray expression values for three representative gene response clusters in control and N-treated experiments. (C,E,G) Row mean (per gene)-normalized expression levels of individual genes are plotted for all genes within the three clusters shown in B,D,F (colored lines), together with the centroid for each cluster (black line); lines are used to connect expression levels between accession control and N-treated experiments for visualization purposes. (B,C) A N-induced cluster in all accessions (N,Accession cluster 14, *n* = 28) in which ‘response to nitrate’ is overrepresented (2 genes, *P* = 2.7E-03). (D,E) A N-repressed cluster in all accessions (N,Accession cluster 5, *n* = 8), in which ‘nucleic acid binding transcription factor activity’ is overrepresented (3 genes, *P* = 8.4E-03). (F,G) A N-regulated cluster with differential induction in accessions (N*Accession cluster 1, *n* = 14), in which ‘cyclin-dependent protein kinase (CDK) inhibitor activity’ is overrepresented (1 gene, *P* = 5.2E-03).

We sought to narrow candidates within genomic intervals that were implicated by SNPs by focusing on potential plasticity regulators that showed variable gene expression among accessions or between conditions or both. Thus, we profiled root gene expression of seven accessions that represent diverse root architectures (Col-0, Kas-2, Var2-1, Tamm-27, NFA-8, Sq-8, Ts-5; Figures 1,2A–C) using ATH1 microarrays in response to a 2-hour treatment of nitrate vs. control to identify early growth regulators that respond to new conditions (Methods; Table S9). ANOVA followed by a model simplification assignment (FDR<0.1, see Methods and Tables S10, S11) identified 5,043 genes that varied among accessions but with no response to nitrogen and 279 genes with a range of effects due to nitrogen (Figure 3). Of these 279 genes, 29 genes responded to nitrogen in all accessions with no accession-specific variation in the degree of response or direction of nitrogen-regulation (“nitrogen-only effect”). 123 genes responded to nitrogen across all accessions, with the same direction of response in all accessions but with a variation in the degree of response (“nitrogen, accession effect’). The remaining 127 genes had a nitrogen*accession interaction effect whereby the direction and/or degree of nitrogen regulation varied over the seven accessions. To validate the expression analysis for nitrogen responses, we analyzed the 152 genes that responded across all accessions (29 genes with nitrogen effect only; 123 genes with nitrogen and accession effect; Table S11). These 152 core response genes include two key nitrate response genes (*AtNRT2.1* (nitrate transporter 2.1, At1g08090) and *NIR1* (nitrite reductase, At2g15620)) and there is an overrepresentation of the GO term ‘response to nitrogen’ (8 genes, *P* = 1.06E-02). In addition, there is an overrepresentation of a number of metabolic functional terms including GO term ‘cellular metabolic process’ (78 genes, *P* = 3.88E-04) and GO term ‘small molecule biosynthetic process’ (23 genes, *P* = 6.69E-03), supporting common nitrogen regulation of cellular and metabolic pathways.

We also defined a more stringent list of regulated genes following the hypothesis that genes controlling the nitrogen response of root traits across accessions should have varied nitrogen-response levels across accessions. To generate such a “stringent set” of candidate genes, we took genes that showed a significant nitrogen*accession effect in ANOVA (127 genes) and those in expression clusters that correlated with average lateral root length in either low or high nitrogen or the difference between the two levels of nitrogen (321 genes).

We then conducted a reverse genetic screen in Col-0 to ask whether GWAS refined by expression analysis could identify genes that mediate specific traits in specific nitrogen environments. As a proxy for examining the phenotypic effects of natural alleles, we evaluated T-DNA mutants in 13 genes that fit two criteria for predicting a specific phenotype: the genes were found within genomic intervals associated with lateral root average length and their transcripts demonstrated a significant ANOVA effect (accession-only, nitrogen or nitrogen*accession effect) among the seven profiled accessions (13/53; Table S8).

### *JR1* and *UBQ14* mediate nitrogen and root trait specific regulatory controls

**Figure 4:**
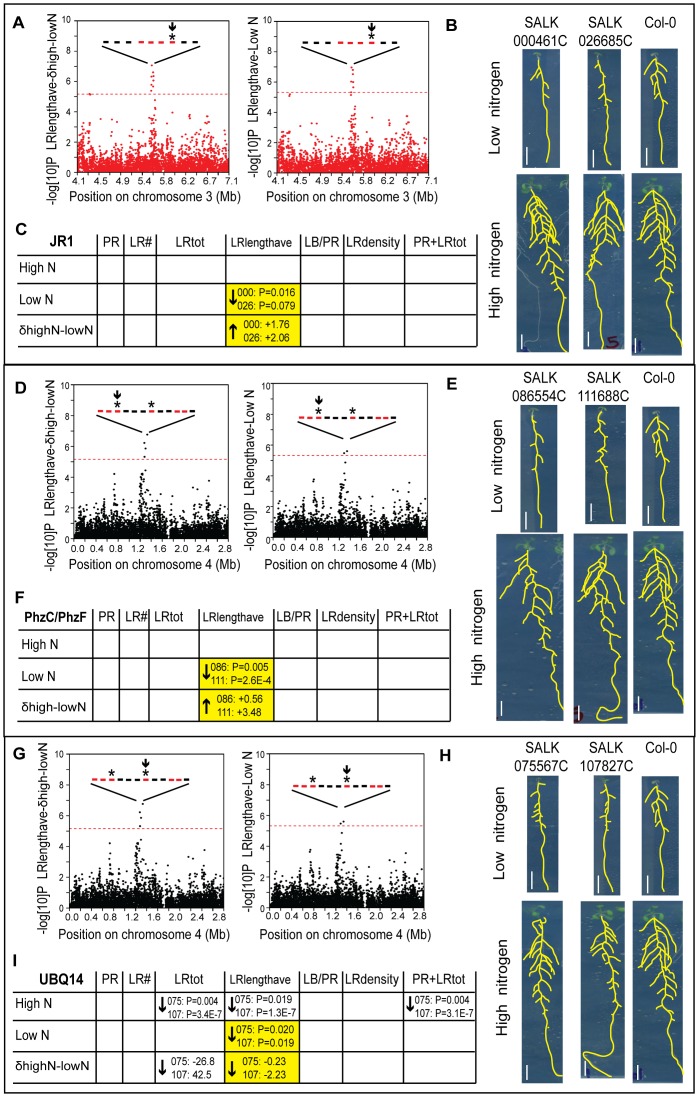
Functional validation of root architecture regulators *JR1* (A–C), *PhzC/PhF* (D–F), and *UBQ14* (G–I). (A,D,G) Genome-wide *P* values from the GWAS magnified around SNP hits; horizontal dashed line corresponds to the 5% FDR threshold that corrects for multiple simultaneous tests. Genes within the 20 Kb boundary around the SNP hits are represented with bars; red color denotes genes whose expression changes in response to nitrogen or among accessions (Table S11), asterisks denote mutants with phenotypes, arrow denotes the gene described in each panel. (B,E,H) Images of four 12 day old seedlings grown on low or high N for two T-DNA alleles in each gene and Col-0; scale bars = 1 cm. (C,F,I) GWAS prediction on trait phenotypes (yellow) and observed phenotypes from confirmed T-DNA alleles indicated with arrows with *P* value, where arrow denotes direction of change in mutant vs. wild type and *t*-test *P* value shows significance of the trait difference in mutant vs. wild type (denoted with first three SALK digits); see Table S12.

Out of the 13 loci, three genes passed our criteria for demonstrating root phenotypes with (1) consistent, quantifiable phenotype in specific root traits for two separate T-DNA mutant alleles and, (2) absent or reduced gene expression in the mutant gene (Figure 4; Methods; Table S12; Figure S10). In addition we carried out crosses of the pairs of allelic mutants and confirmed trans non-complementation, supporting that the mutant alleles were responsible for the phenotypes (Table S12). In support of a model in which genes control traits in specific environments, mutant phenotypes from two loci precisely matched predictions for mediating specific traits in specific environments. One GWAS hit included a block of genes containing *JR1* (*JASMONATE RESPONSIVE 1*), which was associated with lateral root length in low nitrogen and the difference between low and high nitrogen (Figure 4A). In addition, *JR1* met stringent expression criteria, belonging to a cluster that correlated to the difference in lateral root length between nitrogen environments (Figure S9). The two mutant alleles tested showed a specific defect in lateral root average length in low nitrogen but not high nitrogen, as we predicted from analysis of GWAS and expression data (Figure 4B–C). In one plausible functional role for *JR1* in controlling the length of lateral roots specifically when there are low levels of nitrogen in the environment, the jasmonate pathway has been shown to have a role in lateral root development [20]. A second gene, *PhzC*, which was significantly regulated across accessions, was also consistent with GWAS predictions, having shorter lateral roots on low nitrogen but not high nitrogen (Figure 4D–F). For a third gene identified from GWAS analysis, *UBQ14* (polyubiquitin), an association with lateral root length in low nitrogen but not high nitrogen was tested (Figure 4G). However, phenotypic analysis showed phenotypes in both nitrogen conditions. In addition, phenotypes were observed in total lateral root length (LRtot) and total root length (PR+LRtot), as might be expected with severe defects in lateral root length (Figure 4H–I). Thus, this mutant implicates *UBQ14* in trait specificity but not environmental specificity. Nonetheless, for two out of three cases for which we identified a root trait phenotype (*JR1* and *PhzC*) the combination of GWAS with expression profiling identified genes that affected specific traits in specific environments, showing that, with this combination of techniques, we can map genotype to both trait and environment.

Within the narrower set of only three candidate genes that met the dual criteria of belonging to the gene expression “stringent set” and a GWAS “group,” two genes showed mutant phenotypes in precisely the predicted trait and environment. We cannot rule out that expression criteria alone could have identified candidates with mutant phenotypes. However, in a preliminary screen on the same data, we used expression criteria to examine mutants in 13 genes, with some showing pleiotropic phenotypes (data not shown) but none demonstrating specific defects in one or even two traits. Thus, we believe the combination of genome-wide association and gene expression greatly assists in identifying genes involved in specific traits in specific environments with high precision.

Overall, mutations in two out of the three loci that we identified by GWAS affected root systems in low nitrogen environments, where the lateral root system was relatively small, but showed normal root length in high nitrogen environments, where the root system was more extensive. This suggests that the mutant phenotypes are not simply due to general defects in lateral root growth, but rather the gene's specific role in one environment. We point out that we do not know the causal polymorphisms for the phenotypic variation in root traits among natural variants. Even if causal polymorphisms map to the same loci as the mutations we identified, the genetic polymorphisms responsible for the trait variation likely control trait values in a different manner than loss-of-function mutations. However, the mutant analysis provides some corroboration that these loci contribute to controlling plasticity in the traits that we identified. Furthermore, the mutant analysis suggests that different mechanisms may predominate in the control of specific traits in specific environments, perhaps because fewer redundant mechanisms are expressed in one condition. Genotype x environment effects have traditionally been seen as a detriment to crop breeding programs, although there is growing interest in accounting for such effects [17]. Our result suggests that mechanisms that alter traits in specific environments may be quite common. Such genes could be exploited to customize crop phenotypes to a specific environment, such as low nitrogen, without, for example, changing an optimal phenotype in high nitrogen environments.

## Materials and Methods

### Plant material

All seeds were obtained from ABRC (set of 96 ‘Nordborg’ lines, CS22660) [21] or NASC (SALK or SAIL T-DNA lines: SAIL_167_A06, SAIL_658_G04, SAIL_448_B08, SALK_026383(BE), SALK_108492C, SALK_000461C, SALK_026685C, SALK_011676(A), SALK_030620(AI), SALK_020347C, SALK_028332 (BO), SALK_112558, SALK_022578C, SALK_060146, SALK_025883C, SALK_068266(BA), SALK_104906C, SALK_045666, SALK_057714(CF), SALK_123616(BV), SALK_047601, SALK_047837(AS), SALK_059126C, SALK_064966(AP), SALK_075567C, SALK_107827C, SALK_086488C, SALK_121520C, SALK_086554C, SALK_111688C) [22]; Table S8 lists location for each T-DNA line.

### Determining growth conditions

Our overall goal in preliminary growth experiments was to find conditions that maximized trait differences between high and low nitrogen environments. To carry out this exploratory phase, *Arabidopsis* seedlings were grown on a combination of different levels of carbon (0, 3, 10, 15, 30, 60 mM sucrose) and nitrogen (0, 0.03, 0.05, 0.1, 0.5, 1, 5, 10, 20 mM KNO_3_). For each seedling, primary root length was measured and the number of lateral roots counted; lateral root density was calculated from these two parameters (Table S1, Figure S1). Our previous work [9] showed that high levels of nitrogen induce lateral root primordium development and repress lateral root emergence, resulting in a higher pre-emergent∶emergent lateral root ratio than on low nitrate conditions. As a ratio this is also the case here, although total lateral root numbers on high nitrate are larger (due to the nutrient effect and longer primary roots; overall size effect).

An increasing concentration of nitrate was found to result in increased primary root length, particularly with concentrations of 0.5 mM KNO_3_ or more (Figure S1A). This inductive effect tended to level off at 5 mM KNO_3_, with primary root length remaining fairly constant at 10 and 20 mM KNO_3_. At lower levels of nitrate the primary root was longer with no or low sucrose in the media, but as the nitrate concentration increased this effect was reversed (primary root length was longer on higher sucrose concentrations. This is likely due to a C∶N balance effect [23]. It was also on higher sucrose concentrations that the nitrate inductive effect was more pronounced. A similar C/N effect was found on regulation of lateral root number, and again at more than 0.5 mM KNO_3_ the N effect was most pronounced, leveling off at 5 mM (Figure S1B). At the highest sucrose concentrations (30 and 60 mM sucrose) a significant increase in lateral root numbers was observed. Lateral root density was found to be relatively constant over all C∶N conditions, suggesting that in general, increases in lateral root number were proportional to primary root length (Figure S1C). However, there was a higher lateral root density for combinations of the highest C∶N levels (30,60 mM sucrose∶5,10,20 mM KNO_3_), suggesting that at these concentrations there is a developmental effect that leads to larger numbers of lateral roots developing. Thus, in order to understand the genetic basis of this developmental effect we decided to use 30 mM sucrose, 5 mM KNO_3_ as our ‘high N’ condition; on this combination there also appeared to be strong and near-maximal induction of primary root length and lateral root development (as indicated by the leveling off described above). As a comparative low N condition we decided to use 0.03 mM KNO_3_ (also at 30 mM sucrose) since root growth and development was significantly different from seedlings grown on 5 mM, and the plants would be N-depleted/starved but still viable and growing (compared to 0 mM KNO_3_, complete N starvation). To confirm that the root architecture difference that we observed were due to the effect of different nitrate levels rather than potassium levels we grew Col-0 seedlings on either 5 mM KNO_3_, 5 mM CaNO_3_, or 2.5 mM KNO_3_, 2.5 mM CaNO_3_ and found no major differences between overall root architecture (Table S2, Figure S2). Finally, we have some evidence that the root architecture observed in our chosen conditions correlates with that in field conditions, for example Var2-1 is found in sandy regions and exhibits a highly elongated primary root with very few lateral roots as seen in our experiments (see Figure 1).

### Plant growth and treatments

For phenotypic analysis, seeds from each of 96 *Arabidopsis thaliana* accessions [18] or T-DNA lines were grown on vertical agar plates containing custom nitrogen and sucrose-free 1× Murashige and Skoog basal medium (GibcoBRL, Gaithersburg, USA) supplemented with 30 mM sucrose and either low (0.03 mM) or high (5 mM) KNO_3_ with 0.8% agar (pH 5.7). To confirm the effect of nitrate, KNO_3_ was replaced with CaNO_3_ for Col-0. For microarray studies 6,000 seeds (per replicate, in triplicate) of each accession (Col-0, Kas-2, Var2-1, Tamm-27, NFA-8, Sq-8, Ts-5) were sterilized and sown on liquid 1× Murashige and Skoog basal medium containing no nitrogen or sucrose supplemented with 3 mM sucrose and 0.5 mM ammonium succinate for hydroponic growth as previous [9]. Plants were grown for 12 days in 16 hr light (50 mmol photons m^−2^ s^−1^ light intensity)/8 hr dark cycles at 22°C in growth chambers. For determining growth conditions and for phenotyping of the 96 accessions, 10 seedlings were measured for one replicate of each condition/accession in New York in a Percival growth cabinet (Percival Scientific Inc., Perry, IA,). For T-DNA allele phenotyping, an average of 10 seedlings were measured for each of three independent replicates of each condition/allele: New York, T-DNA phenotyping Rep 1 in a Percival Scientific Inc; Warwick, T-DNA phenotyping Reps 2,3 in a Sanyo MLR-351, Panasonic Biomedical, Loughborough). To confirm presence of T-DNA insertions and loss-of-expression of candidate genes, roots were harvested for genotyping of isolated DNA and qPCR of isolated RNA (see Table S12). For treatments, KNO_3_ was added to the media to a final concentration of 5 mM for two hours [9]. Control plants were mock-treated by adding the same concentration of KCl. At the end of the two hour treatment, roots were harvested and flash-frozen in N_2_(l) for subsequent RNA extraction. To confirm trans non-complementation among alleles for each gene, we crossed the pairs of alleles to each other via reciprocal crossing. As a crossing control, individual alleles were also crossed to Col0. Root phenotypes in the F1s were compared to selfed Col0 plants grown in parallel.

### Phenotypic analysis and clustering

In each KNO_3_ environment, parameters relating to root architecture were measured using ImageJ: primary root length (i, PR), number of lateral roots (ii, LR#), lengths of all LRs and LR distribution (number of LRs per cm of PR). From this the following were calculated: lateral root density (iii, LRdensity), the proportion of the PR that is the root branching zone (the zone of the parent root that extends from the most rootward emerged LR to the shoot base, L_B_, terminology following Dubrovsky and Forde (2012) [24]) (iv, L_B_/PR), total LR length (v, LRtot), total LR plus PR length (vi, PR+LRtot), average LR length (vii, LRlengthave); Table S3, Figure S3. Traits designated with roman numerals were used for GWAS. Shoot area was estimated to calculate shoot area to primary root length. Data was scaled from 0 to 1 using the scaling factor (n - low val)/(high val – low val); Table S4. Clustering of phenotyping values was carried out using hierarchical clustering with an average linkage and Pearson correlation using the clustergram function in MATLAB (The MathWorks, Natick, MA, USA). NA values were considered to have a value of 0. Silhouette widths were plotted in MATLAB using the silhouette function for each hierarchical tree and used to determine where to cut the trees and define clusters. A Perl script was written that produces a line drawing illustrating average seedling PR length, and lengths and distribution of LRs in each cm of the PR. This script can be accessed via URL: http://coruzzilab.bio.nyu.edu/cgi-bin/manpreetkatari/drawplant/drawplant.cgi. A positive hit in the reverse genetic screen was determined by satisfying the following criteria: (1) two separate mutant alleles showed the same phenotype, (2) mutant alleles showed a reduction or complete loss of expression using qPCR, 3) both mutant alleles showed a consistent, quantifiable phenotype in three independent screens including separate trials in New York and Warwick growth facilities.

### Calculation of trait heritabilities

To calculate heritabilities of the within-environment variables, we used the “lmer” function of the lme4 package [25] in R v.2.15.1 [26] and fit a restricted maximum likelihood (REML)-based analysis of variance (ANOVA) model of the form: Phenotype = Accession+Error, where Accession was treated as a random effect [27]. Heritabilities were calculated as σ_G_/σ_P_, where σ_G_ is the genetic variance component (the genetic variance component attributable to variation among accessions) and σ_P_ is the total phenotypic variance. To calculate the heritabilities of the response variables, we used the same function in R to fit a REML-based ANOVA model of the form: Phenotype = Accession+Nitrogen Level+Accession-by-Nitrogen Level+Error, where Nitrogen Level (high or low) was treated as a fixed effect, and Accession and Accession-by-Nitrogen Level were treated as random effects. Heritabilities were calculated as σ_GxE_/σ_P_, where σ_GxE_ is the variance component of the Accession-by-Nitrogen Level interaction effect [28].

### Genome-wide association study (GWAS)

GWAS was carried out using the EMMA package in R as described in Atwell et al (2010) [19]. The kinship matrix was constructed using the full set of ∼214k SNPs and SNPs with a minor allele frequency of 0.1 were mapped (∼178k/214k SNPs); see Figure S7. We opted to avoid what we believe is the overly stringent criteria of the Bonferroni correction and adjusted for multiple testing following Moran (2003) and Storey and Tibshirani (2003) [29], [30]. Thus, we calculated *Q*-values, in which the distribution of *P*-values is used to correct for the false positive rate [30]. *Q*-values were calculated separately for each trait using the “qvalue” package [31] in R, a well-established method for finding significant fold changes in the microarray literature (e.g. [32]). We note that, because *Q*-values are based on the distribution of the raw *P*-values and because *Q*-values are calculated separately for each trait, the raw *P*-value corresponding to our target threshold of *Q* = 0.05 (the significance threshold for SNP-trait associations) varies for different traits (see Figure S7). Compared to Atwell et al (2010) [19] we used a more stringent location criteria for selection of genes: for each significant SNP association, a window of 20 kb (rather than 40 kb) centered on the SNP (using the SNP-mapped TAIR8 genome version) was used to select genes predicted to be responsible for the association.

### GWAS power analysis

To understand the relationship between minor allele frequency (MAF), additive genetic effect size, and the power to detect an additive genetic effect, we performed a power simulation *sensu* Yu et al. (2006) [33]. Specifically: (i) the empirical phenotypic values for each accession were treated as random deviates; (ii) based on the empirical phenotypic variation, we calculated a genetic effect equal to 0.1, 0.2, 0.5, 0.7, 0.9, or 1 times the standard deviation of the phenotypic mean; (iii) x accessions out of the total n accessions were randomly assigned to one simulated genotype, and the rest of the individuals were assigned to the other simulated genotype, so that x/n equaled the minor allele frequency of interest; (iv) the genetic effect corresponding to an accession's simulated genotype was added to the empirical phenotypic value for that accession; (v) structured association mapping was performed using the real (non-simulated) kinship matrix; (vi) steps 1 to 4 were repeated 1000 times, and the power to detect the additive genetic effect was the proportion of times that the *P* value from the mapping analyses (see step v) was below the 0.05 significance threshold. We performed this power simulation for each trait and for MAFs ranging from 0.1–0.5; see Figure S8. The power simulations show similar results to Yu et al. (2006) [33], namely that the power to detect a genetic effect is low at small MAFs and at small genetic effect sizes; the power to detect a genetic effect increases dramatically with an increase in the genetic effect size, such that a genetic effect half as large as the random background variation will usually be statistically significant even at a low MAFs.

### RNA isolation, qPCR and microarray experiments

RNA from the whole root samples for microarray analysis was extracted with TRIzol (Invitrogen, Carlsbad, CA). Standard Affymetrix protocols were then used for amplifying, labeling and hybridizing 1 µg of RNA samples to the ATH1 GeneChip (Affymetrix, Santa Clara, USA). For qPCR tests, RNA was extracted with the RNAeasy kit (Qiagen) then first DNAase-treated using a Precision DNase kit and double stranded cDNA was synthesized using the nanoscript RT kit (both from Primer Design Ltd, Southampton, UK) according to manufacturer's instructions. qPCR was carried out using the Precision-SY MasterMix kit using primers designed by Primer Design Ltd according to manufacturer's instructions on a Roche 480 LightCycler. The mRNA levels were normalized relative to the *UBQ10* housekeeping gene using the geNorm REF gene kit (Primer Design Ltd) and quantified using standard curves generated for each primer pair. Expression of At3g16470, At4g02860 and At4g02890 transcripts were used to confirm loss-of expression in the SALK lines vs. Col-0 (primers designed by Primer Design Ltd). SALK lines were PCR-genotyped with primer designed using T-DNA Primer Design (http://signal.salk.edu/tdnaprimers.2.html); for all primer sequences see Table S12.

### Microarray expression normalization and filtering

Affymetrix GCOS software was used to verify that the arrays had similar hybridization efficiencies and background intensities for all accessions. We carried out an analysis to address the use of the Affymetrix Col-0 chip for other *Arabidopsis* accessions. Given the rate of SNPs between accessions and Col-0, we first observed that mismatches to any of the 11 probes (25mers) for any given gene were likely to be rare. In addition, we compared only N-deplete with N-replete Affymetrix signal values within each accession directly and only focused on genes that showed a difference between the two. Therefore any genes that cannot be detected because of sequence-associated probe hybridization problems do not confound our analysis. However, to ensure that we account for over/under-estimations of N-regulation significance that might result from stronger hybridization of a sequence in one accession compared to another (due to sequence difference), we developed an algorithm to rank the signal values of each element in each probe set across the experiments (7 accessions, 2 conditions (N-treatment and KCl control), 3 replicates). This was based on the expectation that, while overall signal from the probe sets of a given gene may change, the relative hybridization to each probe set for a given should not. The method identifies elements within probe sets whose expression is indicative of that element not hybridizing to accession-derived sequences due to the presence of SNP(s) using Col-0 as a reference. Significant deviation from this order could indicate sequence divergence altering the binding strength of a sequence to a probe element. We derived a null distribution of signal strength orders for Col-0 and then used this to identify significant probe element outliers in hybridizations from the other (see Table S9 for lists of all element outliers). These probe elements were discarded. Microarray data was subsequently normalized with MAS5 using all but these element values and implemented in the Affymetrix GCOS software (Table S10). On average, 10% of all probe sets were analyzed with the complete set of 11 elements and a further 70% analyzed with 9 or 10 probe elements (see Table S9 for details for each replicate set). The reproducibility of replicates was analyzed using the correlation coefficient and *r^2^* value of replicate pairs in R; *r* ^2^ values were typically in the range of 0.92 to 0.98, with the lowest being 0.91. Probe-gene mapping was made using the latest annotation file (TAIR10 annotation) (ftp://ftp.arabidopsis.org/home/tair/Microarrays/Affymetrix/affy_ ATH1_array_elements-2010-12-20.txt). The following classes of probes were flagged (Table S10): probes matching non-nuclear *Arabidopsis thaliana* genes or that had no gene match (flag #1), probes that had an ambiguous match to nuclear genes, i.e. matched more than one gene (flag #2), probes where several probes match a single gene (flag #3), probes whose average expression level was found to be below the detection cutoff (flag #4). To identify flag #4 probes we analyzed genes known to be expressed or absent in the root to calculate an expression signal of 100 as a cutoff for detection.

### Expression ANOVA, statistical analysis and clustering

All genes were fit to the following ANOVA model: Y = μ+α_accession_+α_treatment_+α_accession* treatment_+ε, where Y is the normalized signal of a gene, μ is the mean of the reference accession and treatment (intercept), the α coefficients correspond to the effects of accession, treatment (nitrogen) and the interaction between accession and treatment, and ε represents unexplained variance. Potential location effects were handled by growing plants together in a highly controlled environment and randomizing the placement of accessions and treatments in different shelves and locations of the growth chamber. The replicate trials were conducted in rapid succession in identical conditions, where we have not found significant time-effect variation. Thus, the ANOVA was modeled without block effects, where potential confounding effects were handled by randomization. Genes with a model *P* value less than a cutoff determined by setting the FDR [34] to 0.1 were analyzed further using model simplification to test these genes for significant N*Accession interaction effects, response to N, and variation across Accession. We did this by removing terms from the model one by one and then comparing the models to see if there was a significant difference in explanatory power between the simplified model and the more complex model using an FDR of 0.1. Gene expression values were averaged for each treatment, log2 converted, row normalized and clustered using hierarchical clustering with an average linkage and Pearson correlation using the clustergram function in MATLAB. Silhouette widths were plotted in MATLAB using the silhouette function for each hierarchical tree and used to determine where to cut the trees and define clusters. Clustering was carried out separately for genes that were determined by ANOVA to have a N,Accession effect, a N*Accession effect, or a N only effect, then the cluster patterns visualised together in MATLAB using the clustergram function to create Figure 3A. Two-tailed t-tests assuming equal variance were used to compare trait values for wild-type and mutant seedling roots, and trait values for Col-0 grown on different levels of sucrose and nitrate. For analysis of overrepresentation of GO terms we used the BioMaps function in VirtualPlant with default settings [35].

### Mixed model ANOVA

Root phenotypes for the seven transcriptionally profiled accessions (Figure S6) were analyzed using a mixed interaction model ANOVA using MATLAB (anovan function) with the following model: ROOT_TRAIT_n_ = ENV_n_+GEN_n_+GEN_n_ * ENV_n_+e_n_ where Root Trait_n_ is one of n = 7 root traits measured (PR, LRtot, PR+LRtot, LRlengthave, LR#, LRdensity, and L_B_/PR), ENV is environment, GEN is genotype, and e is error. Environment was modeled as a fixed effect and genotype was modeled as a random effect. *P* values were taken for each trait separately for the main and interaction effects. Coefficients generated from the ANOVA were used to determine the specific traits that contributed most to significant interaction effects (Table S5). As in the design for expression analysis, placement of plants was randomized in chambers, and this experiment was conducted at one time point.

### Principal Components Analysis

Principal Components Analysis was performed in MATLAB using the princomp function with default parameters. Rows were accessions and traits were columns, where dimensionality reduction was performed on traits. The biplot function was used to map accessions in specific treatments (average trait values) in the new trait space and observe the contribution of original traits to each new component. We performed separate analyses on the combined HighN and LowN treatments for each accession, each condition alone, and δ highN-lowN of each accession to changes in nitrogen. We plotted two components on each biplot (1 vs 2; 2 vs 3) to analyze the first three principal components. See Figure S5.

### Network of expression modules and traits

We created a network of expression modules to traits by first clustering responses (expression in low nitrogen – expression in high nitrogen) using Pearson correlation and hierarchical clustering (average linkage, tree cut at R = 0.7). To determine significant clusters, we randomized the data and used the same clustering routine. This routine showed that clusters greater than 50 genes were observed less than 10% of the time by chance. Using that cutoff to define major clusters, we took the mean response of these major clusters in all 7 accessions. We then concatenated mean scaled trait values for δ highN-lowN and generated a correlation matrix, where R>0.7 or <−0.7 resulted in a significant edge. This resulted in a correlation matrix between gene expression clusters and traits that was used to generate the network depicted in Figure S9 using the biograph function in MATLAB.

## Supporting Information

Figure S1 Nitrogen and sucrose-regulation of root architecture. Seedlings were grown for 12 d on different combinations of varying concentrations of sucrose (from 0 mM to 60 mM) and KNO_3_ (from 0 mM to 20 mM). (A) Average primary root; error bars represent SE, n = 20. (B) the number of lateral roots, and (C) lateral root density was calculated; *n* = 20.
(TIF)

Figure S2 Comparison of CaNO_3_ and KNO_3_ effects on *Arabidopsis* root architecture. Col-0 seedlings were grown for 12 d on basal MS media supplemented with an equal concentration of NO_3_ in the form of either (A) 5.0 mM KNO_3_, (B) 2.5 mM KNO_3_/2.5 mM CaNO_3_, or (C) 5.0 mM CaNO_3_. Scale bar = 1 cm.
(TIF)

Figure S3 Histograms of the distribution of root trait values over the 96 accessions. For high N (A) and low N (B) the following trait distributions are plotted: PR (cm), LRtot (cm), PR+LRtot (cm), LRlengthave (cm), LR#, LRdensity, and L_B_/PR.
(TIF)

Figure S4 Changes in root traits among natural variants between low and high nitrogen environments. The scaled difference between root trait values on low and high nitrogen (δ highN-lowN) is represented in the form of a heatmap (A); see color scale bar for scaled trait value. and trait differences were used to form a dendrogram of accessions resulting in nine clusters as indicated by horizontal lines (B); the seven accessions chosen for expression analysis are highlighted in red.
(TIF)

Figure S5 Principal Components Analysis (PCA) of root trait data. The first three PCs are shown for root traits on low N capturing 97% of the variation (A–B), high N capturing 96% of the variation (C–D), and the combined low N and high N data N capturing 93% of the variation (E–F). Red markers indicate position of 96 accessions as determined by their trait values in the given principal components. Blue lines represent vectors that quantify the magnitude and direction of a trait's contribution to that axis.
(TIF)

Figure S6 Average trait values on low or high N for the seven accessions. PR (cm), LRtot (cm), L_B_/PR, PR+LRtot (cm), LR#, LRlengthave (cm) and LRdensity; error bars represent standard error.
(TIF)

Figure S7 Manhattan plots generated from GWAS analysis for the root traits across the genome. The five chromosomes are distinguished by color. The red horizontal dashed line corresponds to the 5% FDR threshold that corrects for multiple simultaneous tests; this threshold is different for different traits (see Methods).
(TIF)

Figure S8 Power analyses for the 27 trait/environment combinations measured for the 96 accessions. Average power is plotted against genetic effect for traits predicted to have a minor allele frequency of 0.1, 0.2, 0.3, 0.4 and 0.5.
(TIF)

Figure S9 Network mapping gene expression N responses to trait δ highN-lowN differences. Edges are drawn where there is a correlation of R>0.7 or <−0.7 between expression N response clusters and the δ highN-lowN of traits across the 7 accessions transcriptionally profiled. Traits are shown in blue-colored boxes. Edges between LRlengthave and expression clusters are colored green and the edge between LRlengthave and the N*Accession ANOVA affect genes colored orange.
(TIF)

Figure S10 T-DNA locations and effect on gene expression for *JR1*, *PhC/PhZ* and *UBQ14*. (A) Schematic to scale showing the location of the two T-DNA alleles for each gene. (B) qPCR quantification of gene expression for *JR1*, *PhC/PhZ* and *UBQ14*. For visualization purposes the expression levels of each gene is scaled relative to the expression in lowN Col0 having a value of 1; data taken from Table S12.
(TIF)

Table S1 Nitrogen and sucrose-regulation of primary root length. Seedlings were grown for 12 d on different combinations of varying sucrose (from 0 mM to 60 mM) and KNO_3_ concentrations (from 0 mM to 20 mM). Average and SE for (A) primary root length, (B) the number of lateral roots, and (C) LRdensity was calculated; *n* = 20.
(XLSX)

Table S2 Comparison of CaNO_3_ and KNO_3_ effects on *Arabidopsis* root architecture. Col-0 seedlings were grown for 12 d on basal MS media supplemented with an equal concentration of nitrate in the form of either (A) 5.0 mM KNO_3_, (B) 5.0 mM CaNO_3_, or (C) 2.5 mM KNO_3_/2.5 mM CaNO_3_. (D) *T*-test *P* values for statistical comparison of root trait data between each combination.
(XLSX)

Table S3 Shoot and root phenotypic trait data for the 96 accessions. Values are shown for growth on high N, low N, and δ highN-lowN. The traits and units where relevant are: estimated shoot area (SA, cm^2^), primary root length (PR, cm), proportion of SA to PR length (SA to PR), total LR length (LRtot, cm), total LR plus PR length (PR+LRtot, cm), average LR length (LRlengthave, cm), number of LRs (LR#), LR density (LRdensity, LRs per cm PR), proportion of the PR that is the root branching zone (L_B_/PR).
(XLSX)

Table S4 Root phenotypic trait data after scaling for clustering analysis. Values are shown for growth on high N, low N, and the δ highN-lowN. Traits are as described for Table S3, excepting the two shoot-related traits (which were not used for clustering or for further analysis following GWAS).
(XLSX)

Table S5 Mixed model ANOVA values. (A) Average unscaled root trait values and standard error values for the 7 transcriptionally profiled accessions. (B) ANOVA *P* values. (C) ANOVA coefficients.
(XLSX)

Table S6 GWAS hits. Marker positions for traits showing a significant association (cutoff of Q<0.05). For each association the marker location, the minor allele frequency of the SNP, the R^2^ value and the *Q*-value (FDR-corrected *P*-value) are provided. The R^2^ value is from a one-way fixed effect analysis of variance modeling the trait as a function of the SNP site plus error. The R^2^ value is calculated as the sum of squares of the model divided by the total sum of squares (the sum of squares of the model plus the sum of squares of the error) [36]. In addition a list of gene AGI IDs for a 20 Kb window around each SNP is listed, with the distance to the SNP (bp) given underneath each gene; the distance is to the 3′ end of the gene for unshaded bp values, and to the 5′ end of the gene for grey-shaded values.
(XLSX)

Table S7 Heritability (H^2^) of each trait on high N, low N, or δ highN-lowN.
(XLSX)

Table S8 GWAS hits organized by genomic location. The 7 SNP marker groups associated with LRlengthave including 53 genes are tabulated together with information about the trait association. Genes whose regulation is significantly different across accessions are marked and T-DNA line numbers given for genes that were carried forward to functional analysis. The three genes with validated phenotypes are highlighted yellow.
(XLSX)

Table S9 List of all element outliers for probes in each replicate set of microarray data.
(XLSX)

Table S10 MAS5-normalised Affymetrix data for all microarray experiments. The table lists all Affymetrix probe IDs, their associated TAIR10 Arabidopsis Genome Initiative (AGI) gene IDs, and flag designations: probes that match non-nuclear-encoded genes or do not match genes (#1) (754 probes), probes that match more than one gene (#2) (1,064 probes), probes where several probes match a single gene (#3) 182 genes; 367 probes), probes whose expression was determined to be below an expression cutoff (#4 (6169 probes) (as described in Methods). Probes that were found to be significantly differentially expressed in different accessions only, by N only, by N plus accession (N,Accession), by an interaction between N and accession background (N*Accession) according to the ANOVA with model simplification (expression ANOVA effect), and cluster numbers for these genes (expression ANOVA cluster number; see Table S11 for further details of these genes) are given. Probes with N-regulated expression levels that were clustered to trait δ highN-lowN values are designated with cluster numbers (trait∶expression cluster number); see Figure S9.
(XLSX)

Table S11 Genes that are significantly differentially expressed according to accession background and/or nitrogen. Lists of genes whose expression was found to be either significantly differentially expressed (using the expression ANOVA followed by model simplification to designate what the effects were) in different accessions only (Accession only), by N only, by N,Accession, by a N*Accession interaction. Cluster numbers for these genes together with the *P* value are provided. Accession and Accession-P, the expression change and *P* value for expression change in specific accession KCl control sample compared to Col-0 KCl control sample. N∶Accession and N∶Accession-P, the expression change and *P* value for expression change in specific accession KNO_3_ sample compared to Col-0 KNO_3_ sample; N and N-P, the sum of the expression change and *P* value for expression change for the N only effect in all accessions; Interaction and Interaction-P, the sum of the expression change and *P* value for expression change for the N main effect in all accessions plus the N∶E interaction term; *P* value, overall ANOVA *P* value of significance; R-squared, *r* ^2^ value for ANOVA test.
(XLSX)

Table S12 Functional validation of trait associations. (A) Expression data from the transcriptionally profiled accessions for *JR1*, *UBQ14* and *PhzC/PhzF*. (B) qPCR analysis of the *JR1*, *UBQ14* and *PhzC/PhzF* genes in their respective T-DNA mutant backgrounds compared to wild type; primer sequences for qPCR and PCR are listed (see also Figure S10). (C) Average and standard error (SE) root trait values measured from 12 day old seedlings for each of the two T-DNA mutant alleles for *JR1*, *UBQ14* and *PhzC/PhzF*, and for Col-0 wild-type plants; *n* for each genotype is listed. (D) *T*-test *P* values from analysis of the differences between trait values in mutant vs. wild type. (E) Trait values δhighN-lowN for all analyzed genotypes. (F) Trait values for F1 seedlings derived from crosses between pairs of alleles for each gene, together with crossing controls (mutant×Col0) and Col0.
(XLSX)
