# Supplementary figures and images for: Plasticity Regulators Modulate Specific Root Traits in Discrete Nitrogen Environments
Source: PLoS Genet. 2013 Sep 5;9(9):e1003760. doi: 10.1371/journal.pgen.1003760 (PMC3764102; doi:10.1371/journal.pgen.1003760)

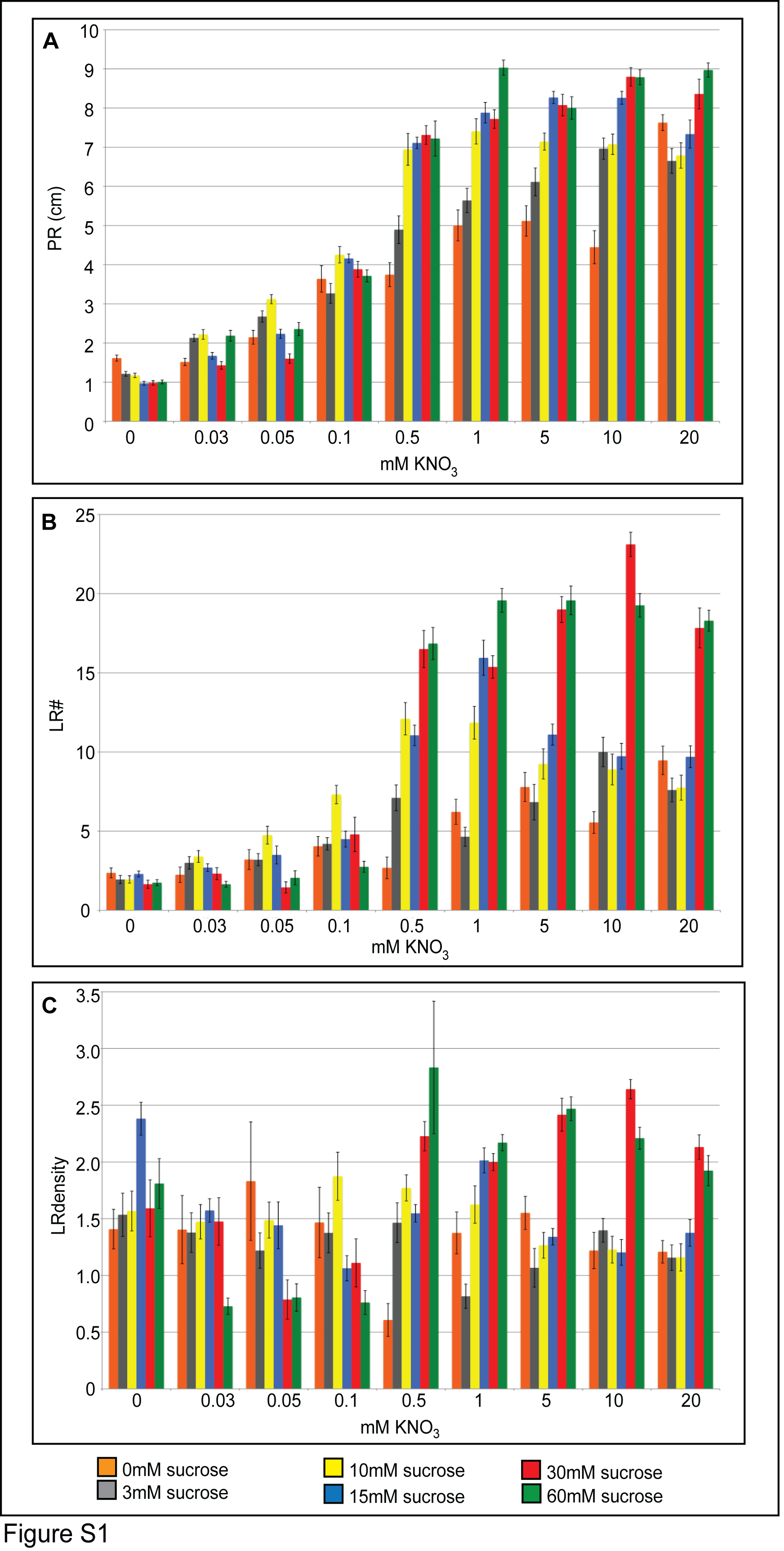

Supplement: Figure S1 — Nitrogen and sucrose-regulation of root architecture. Seedlings were grown for 12 d on different combinations of varying concentrations of sucrose (from 0 mM to 60 mM) and KNO3 (from 0 mM to 20 mM). (A) Average primary root; error bars represent SE, n = 20. (B) the number of lateral roots, and (C) lateral root density was calculated; n = 20. (TIF) [file pgen.1003760.s001.tif]

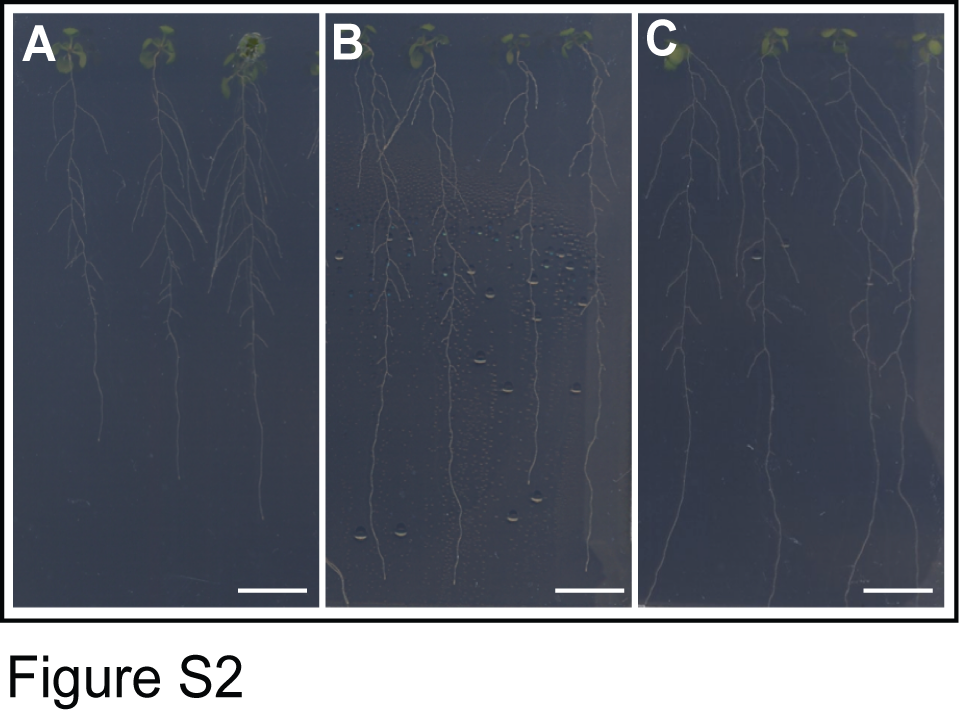

Supplement: Figure S2 — Comparison of CaNO3 and KNO3 effects on Arabidopsis root architecture. Col-0 seedlings were grown for 12 d on basal MS media supplemented with an equal concentration of NO3 in the form of either (A) 5.0 mM KNO3, (B) 2.5 mM KNO3/2.5 mM CaNO3, or (C) 5.0 mM CaNO3. Scale bar = 1 cm. (TIF) [file pgen.1003760.s002.tif]

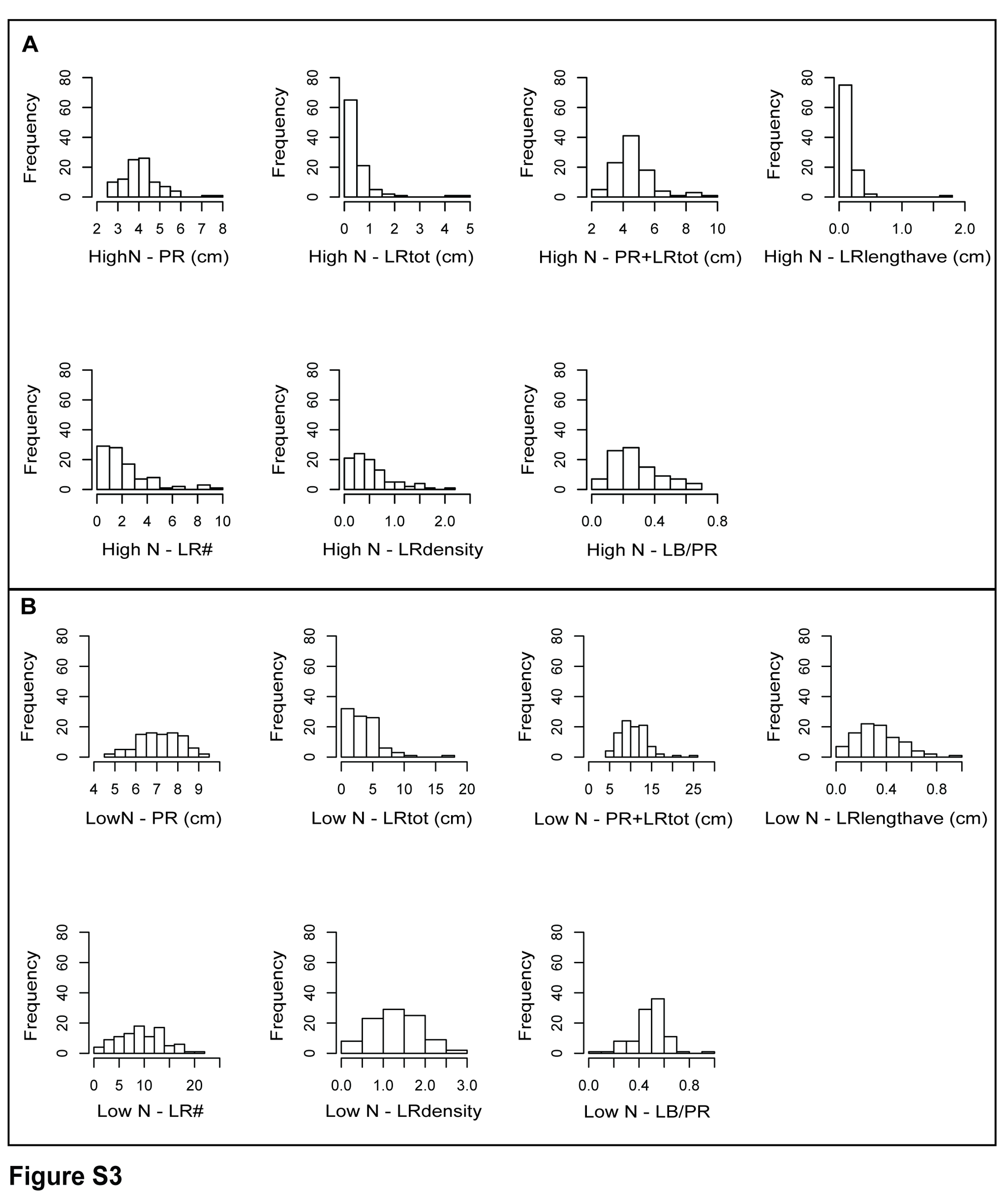

Supplement: Figure S3 — Histograms of the distribution of root trait values over the 96 accessions. For high N (A) and low N (B) the following trait distributions are plotted: PR (cm), LRtot (cm), PR+LRtot (cm), LRlengthave (cm), LR#, LRdensity, and LB/PR. (TIF) [file pgen.1003760.s003.tif]

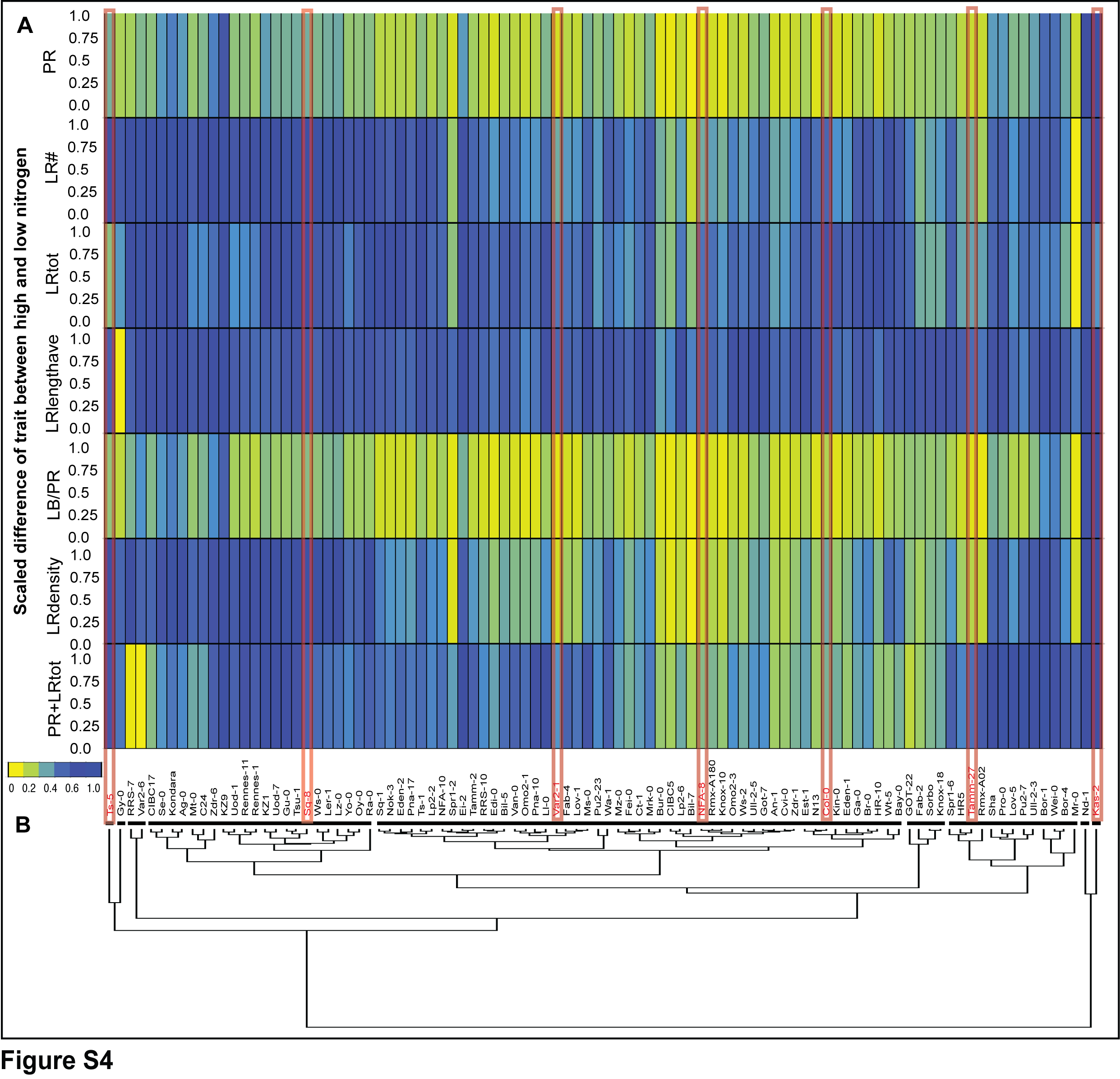

Supplement: Figure S4 — Changes in root traits among natural variants between low and high nitrogen environments. The scaled difference between root trait values on low and high nitrogen (δ highN-lowN) is represented in the form of a heatmap (A); see color scale bar for scaled trait value. and trait differences were used to form a dendrogram of accessions resulting in nine clusters as indicated by horizontal lines (B); the seven accessions chosen for expression analysis are highlighted in red. (TIF) [file pgen.1003760.s004.tif]

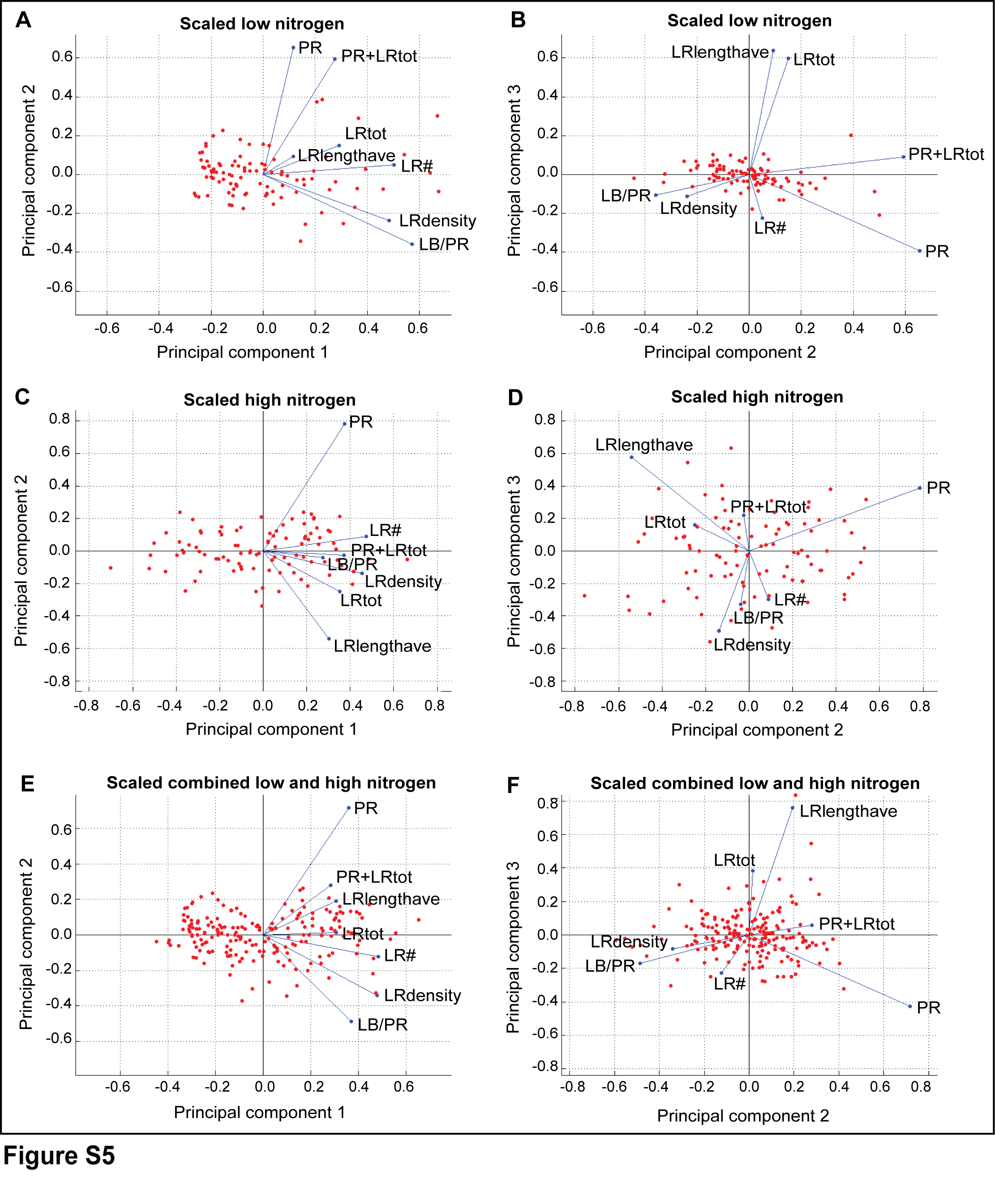

Supplement: Figure S5 — Principal Components Analysis (PCA) of root trait data. The first three PCs are shown for root traits on low N capturing 97% of the variation (A–B), high N capturing 96% of the variation (C–D), and the combined low N and high N data N capturing 93% of the variation (E–F). Red markers indicate position of 96 accessions as determined by their trait values in the given principal components. Blue lines represent vectors that quantify the magnitude and direction of a trait's contribution to that axis. (TIF) [file pgen.1003760.s005.tif]

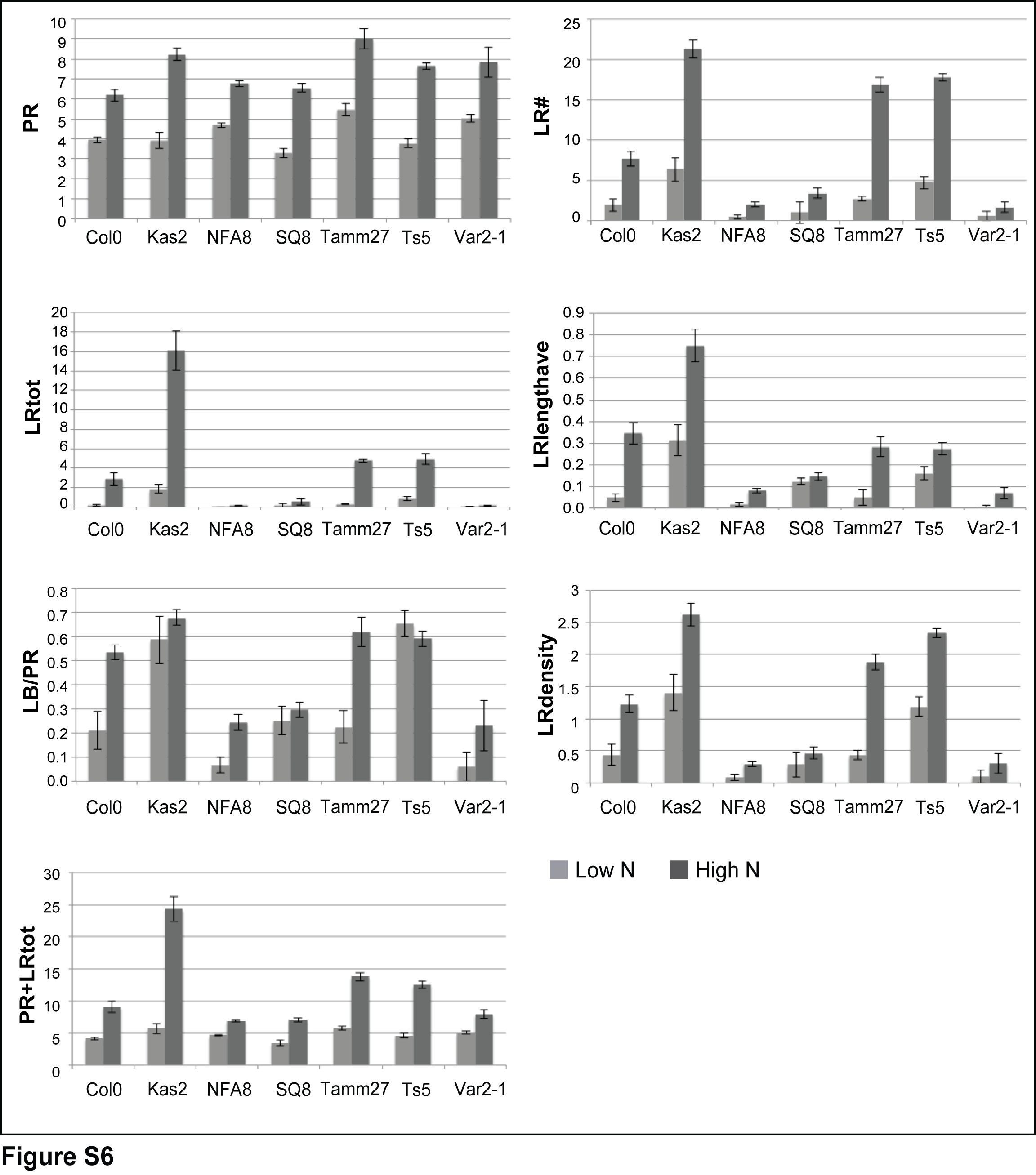

Supplement: Figure S6 — Average trait values on low or high N for the seven accessions. PR (cm), LRtot (cm), LB/PR, PR+LRtot (cm), LR#, LRlengthave (cm) and LRdensity; error bars represent standard error. (TIF) [file pgen.1003760.s006.tif]

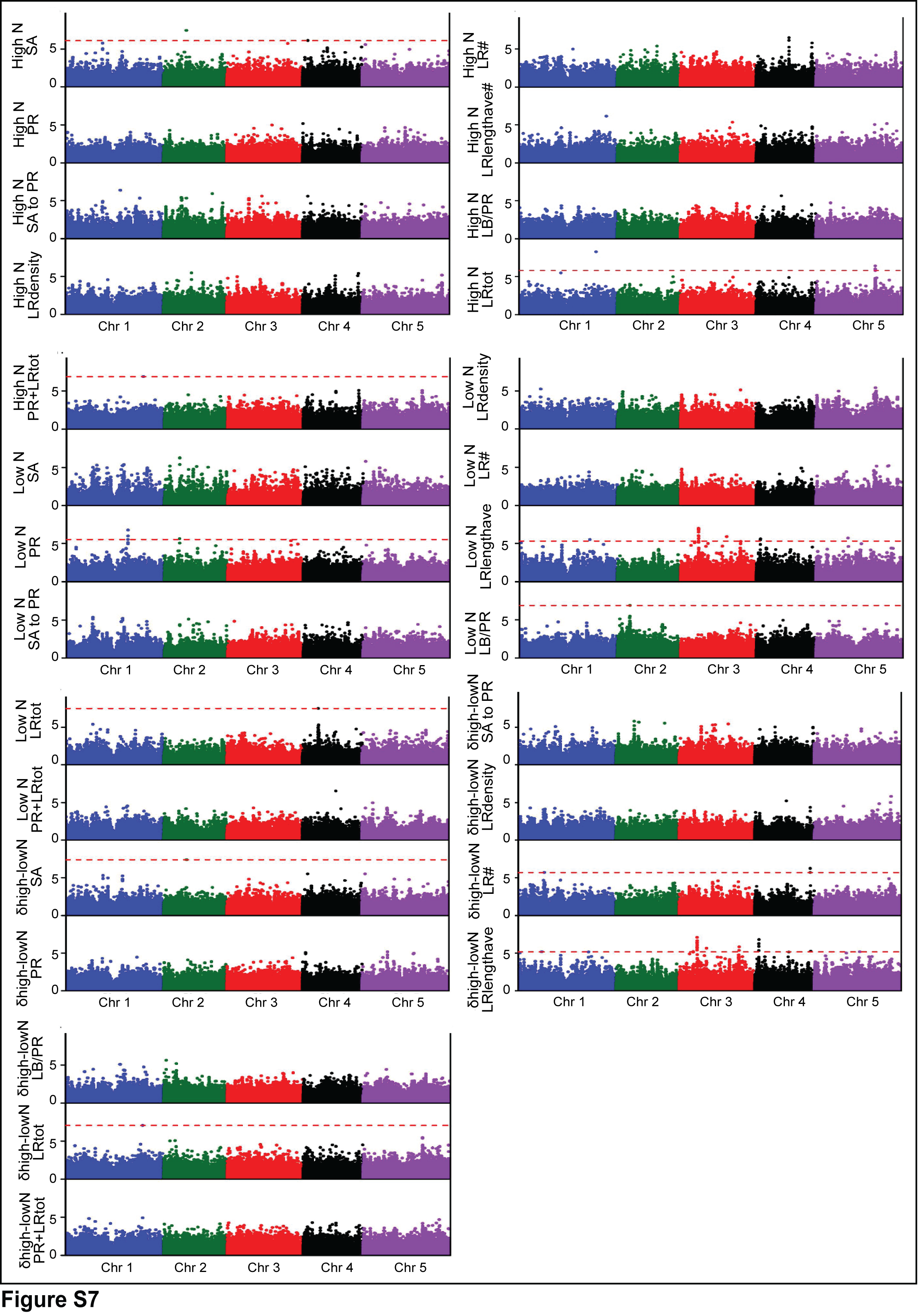

Supplement: Figure S7 — Manhattan plots generated from GWAS analysis for the root traits across the genome. The five chromosomes are distinguished by color. The red horizontal dashed line corresponds to the 5% FDR threshold that corrects for multiple simultaneous tests; this threshold is different for different traits (see Methods). (TIF) [file pgen.1003760.s007.tif]

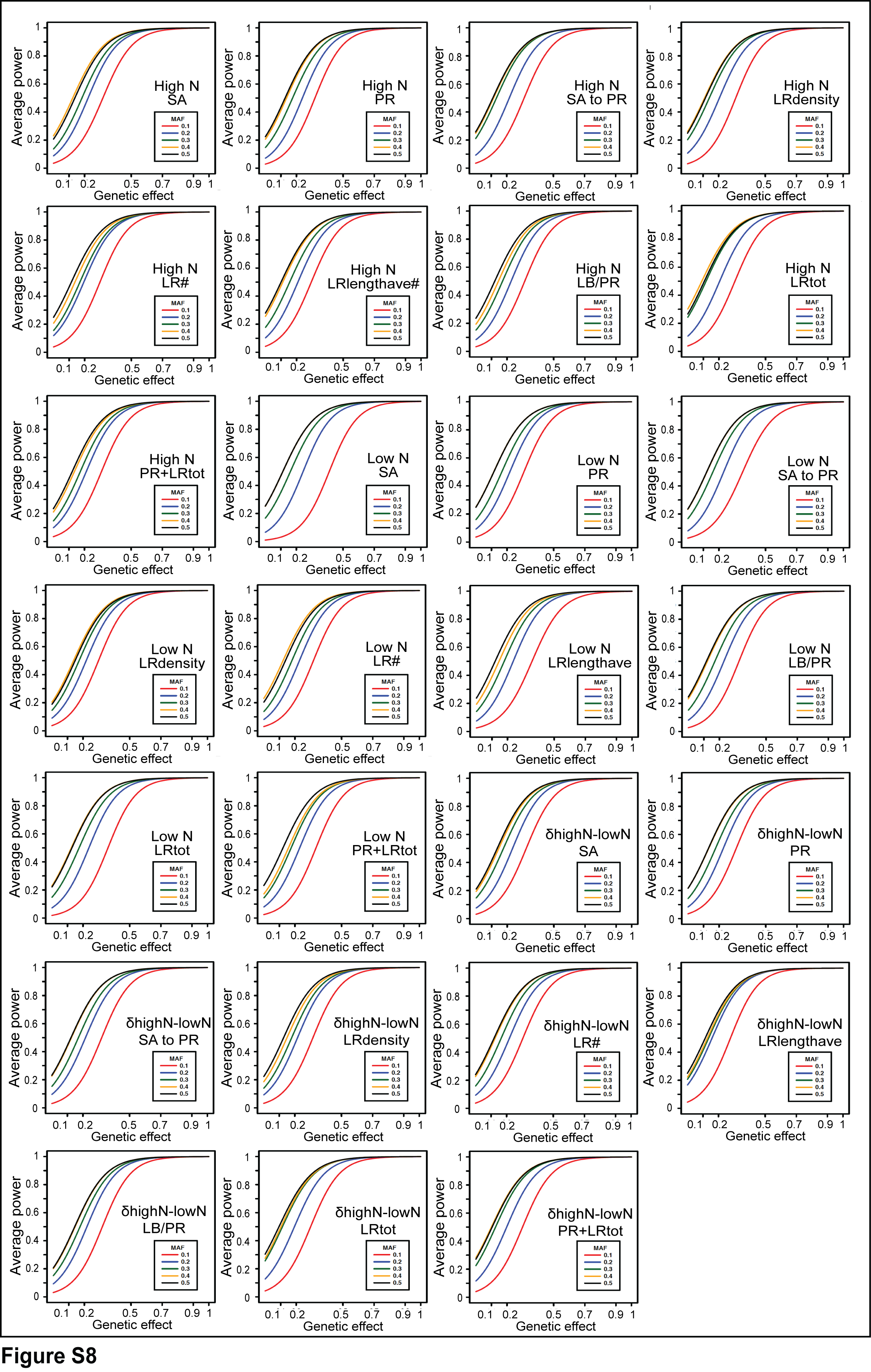

Supplement: Figure S8 — Power analyses for the 27 trait/environment combinations measured for the 96 accessions. Average power is plotted against genetic effect for traits predicted to have a minor allele frequency of 0.1, 0.2, 0.3, 0.4 and 0.5. (TIF) [file pgen.1003760.s008.tif]

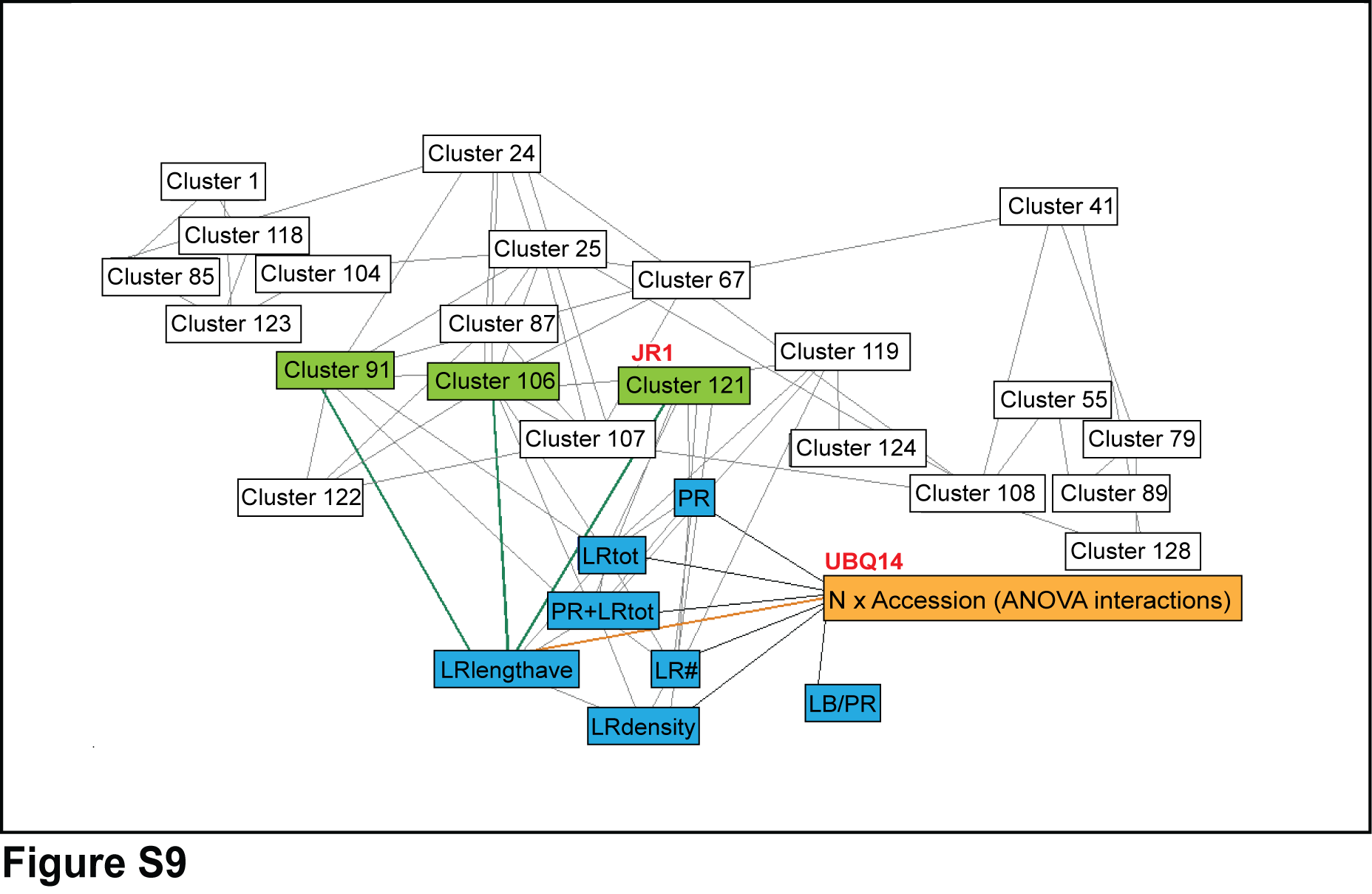

Supplement: Figure S9 — Network mapping gene expression N responses to trait δ highN-lowN differences. Edges are drawn where there is a correlation of R>0.7 or <−0.7 between expression N response clusters and the δ highN-lowN of traits across the 7 accessions transcriptionally profiled. Traits are shown in blue-colored boxes. Edges between LRlengthave and expression clusters are colored green and the edge between LRlengthave and the N*Accession ANOVA affect genes colored orange. (TIF) [file pgen.1003760.s009.tif]

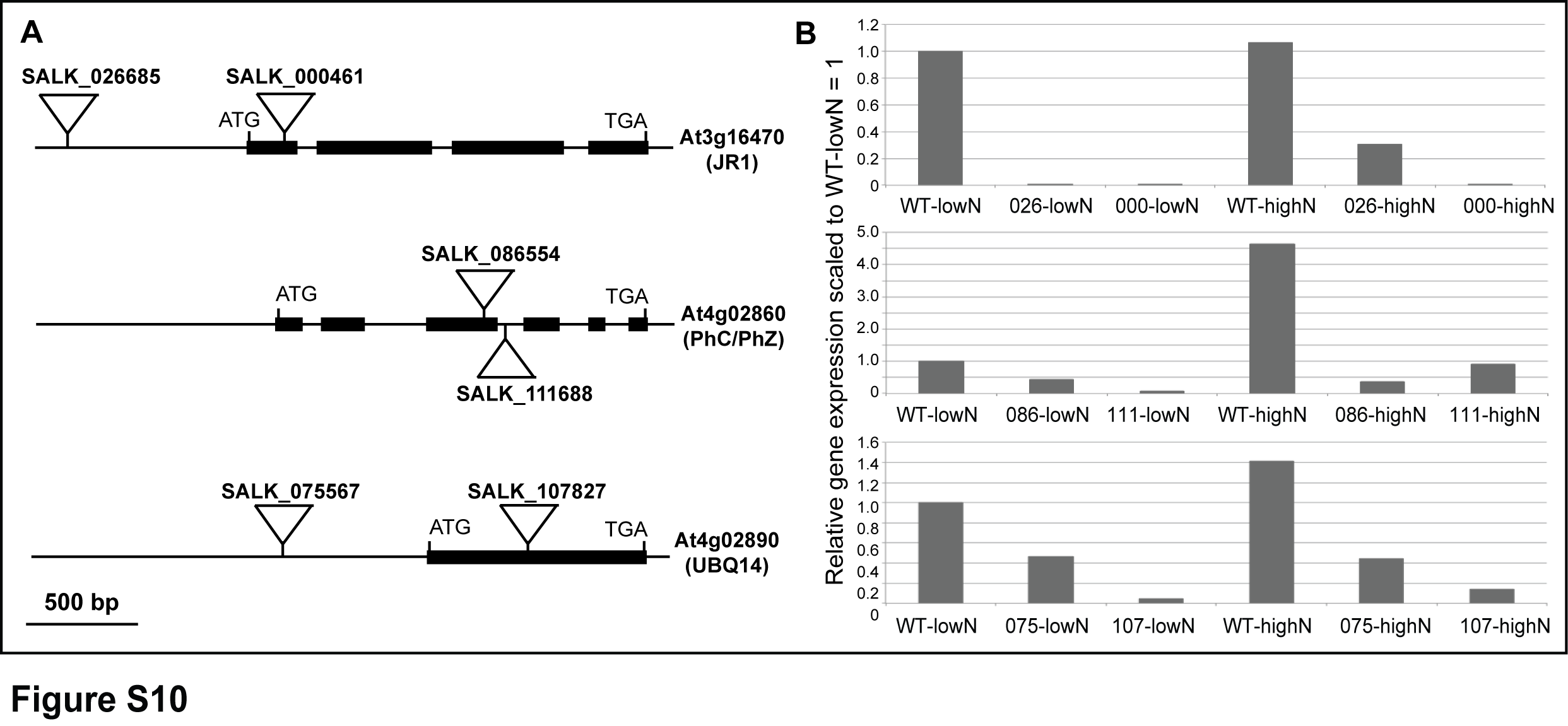

Supplement: Figure S10 — T-DNA locations and effect on gene expression for JR1, PhC/PhZ and UBQ14. (A) Schematic to scale showing the location of the two T-DNA alleles for each gene. (B) qPCR quantification of gene expression for JR1, PhC/PhZ and UBQ14. For visualization purposes the expression levels of each gene is scaled relative to the expression in lowN Col0 having a value of 1; data taken from Table S12. (TIF) [file pgen.1003760.s010.tif]
